# Association of cumulative exposure to Chinese visceral adiposity index and gastrointestinal cancer: a prospective cohort study

**DOI:** 10.3389/fonc.2025.1534682

**Published:** 2025-07-03

**Authors:** Jiaxing Li, Chao Ma, Kuan Liu, Wanchao Wang, Shuqing Cui, Yuan Tian, Zhigang Dong, Wenqiang Wei, Shouling Wu, Siqing Liu

**Affiliations:** ^1^ First Division of Hepatobiliary and Pancreatic Surgery, Affiliated Hospital of North China University of Science and Technology, Tangshan, Hebei, China; ^2^ Graduate School, North China University of Science and Technology, Tangshan, Hebei, China; ^3^ Hepatobiliary Surgery, Affiliated Kailuan General Hospital of North China University of Science and Technology, Tangshan, Hebei, China; ^4^ Health Care Center, Affiliated Kailuan General Hospital of North China University of Science and Technology, Tangshan, Hebei, China

**Keywords:** Chinese visceral adiposity index, gastrointestinal cancer, visceral fat, visceral adipose tissue, cohort study

## Abstract

**Background:**

Using the Kailuan Cohort, we investigated the association between cumulative exposure to the Chinese visceral adiposity index (CVAI) and the risk of developing gastrointestinal (GI) cancers.

**Methods:**

A prospective cohort study was conducted among participants who underwent three consecutive health examinations in the Kailuan Cohort from 2006 to 2010. Participants were categorized into quartiles based on their cumulative CVAI (cumCVAI). The cumulative incidence of GI cancers was estimated using Kaplan-Meier curves. The dose-response relationship between CVAI and the risk of developing GI cancers was examined using restricted cubic spline (RCS) in multivariable adjusted models. Multivariate Cox proportional hazards regression analysis was performed to assess the association between cumCVAI quartiles and the incidence of GI cancers. Furthermore, subgroup analyses and sensitivity analyses were conducted.

**Results:**

A total of 44,213 individuals were included in this study. The incidence rates of GI cancers per 1,000 person-years for the Q1 to Q4 groups were 1.00,1.45,1.62 and 2.11, respectively. The RCS curve demonstrated a significant dose-response relationship between cumCVAI and the occurrence of GI cancers events (*P* for overall trend < 0.001; *P* for nonlinear trend < 0.001). After adjusting for potential confounders, compared with the Q1 group, the risk of GI cancers was significantly elevated in the Q2 group (*HR* 1.26; 95% *CI* 1.01, 1.58), Q3 group (*HR* 1.31; 95% *CI* 1.05, 1.64), and Q4 group (*HR* 1.48; 95% *CI* 1.19, 1.85). This association was particularly evident in older individuals, females, those with a BMI ≥ 24 kg/m^2^, non-smokers, and non-drinkers.

**Conclusion:**

Our findings demonstrate a significant association between high cumCVAI and an increased incidence of GI cancers. Prolonged maintenance of CVAI within optimal levels may serve as a potential preventive strategy for GI cancers.

## 1 Introduction

Gastrointestinal (GI) cancers account for approximately one-quarter of global cancer incidence and one-third of global cancer-related deaths (1). Despite continuous improvements in screening and treatment for GI cancers in recent years, their incidence and mortality rates remain high, and patients often have poor prognoses. As a populous country, China has the highest number of new cases and deaths from GI cancers worldwide (2). Therefore, it is crucial to investigate the risk factors for GI cancers and provide preventive strategies.

Obesity has been established as a risk factor for GI cancers (3),Commonly used surrogate measures of obesity, including body mass index (BMI) (4),waist circumference(WC), waist‐to‐hip ratio (WHpR), waist‐to‐height ratio (WHtR) have been shown to increase cancer risk (5, 6).In recent years, studies have found that visceral obesity is a significant risk factor for cancer development (7),and increases the risk of GI cancers (8, 9).Visceral adipose tissue (VAT), the most direct manifestation of visceral obesity, can more accurately describe fat distribution compared to traditional obesity indicators (10). Clinical assessment of VAT primarily relies on magnetic resonance imaging (MRI) and computed tomography (CT), whose high costs significantly limit their use in epidemiological studies (11). In 2010, a study suggested using the visceral adiposity index (VAI) as a surrogate for VAT, and it showed high concordance with MRI and CT measurements (12). However, the VAI was developed for Caucasian populations and may have limitations in assessing visceral fat distribution in Chinese populations. The Chinese visceral adiposity index (CVAI) was proposed based on the metabolic characteristics of the Chinese population and is a good surrogate indicator of visceral fat distribution in Chinese people (13). Multiple studies have demonstrated that CVAI is significantly associated with diabetes and cardiovascular diseases (14, 15), but its association with GI cancers has been rarely reported.

Previous studies have been limited to single time-point measurements of CVAI, neglecting the longitudinal impact of cumCVAI over time. In this study, using the Kailuan Cohort, we prospectively investigated the association between cumulative exposure to CVAI and the risk of developing GI cancers.

## 2 Methods

### 2.1 Study population

**Figure 1:**
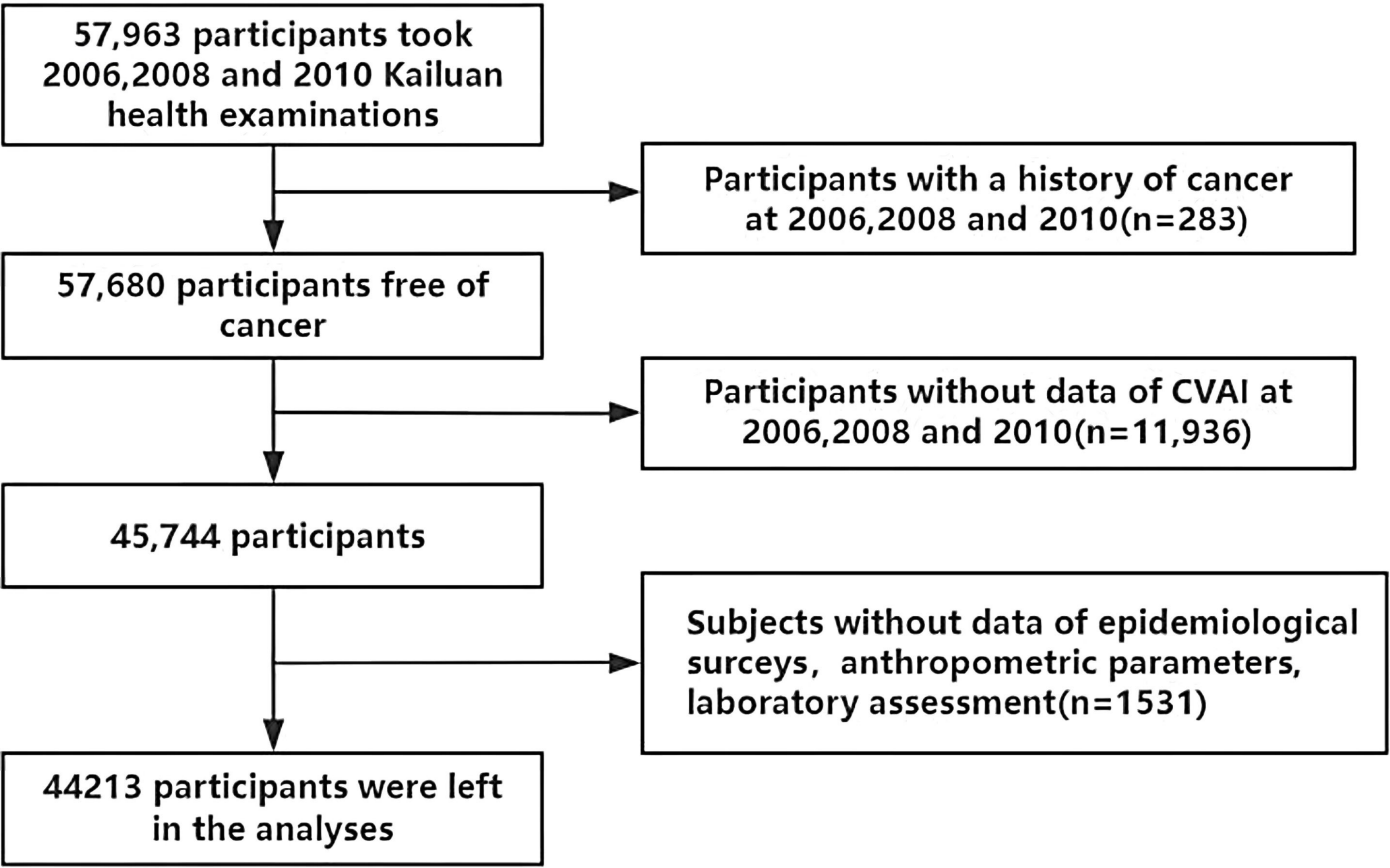
Screening flowchart.

Our study population was derived from the Kailuan Cohort (registration number:ChiCTR-TNC-11001489; https://www.chictr.org.cn/showprojEN.html?proj=48316), which consists of employees (including retirees) of the Kailuan Group. Detailed information about the Kailuan Cohort has been previously reported (16). In summary, the Kailuan Cohort was established in 2006 by recruiting 101,510 employees (aged 18–98 years) from the Kailuan Group. Participants underwent a series of physical examinations, biochemical tests, and questionnaires at 11 affiliated hospitals of the Kailuan Group. Subsequently, these assessments were repeated every two years, resulting in eight waves of data collection to date. Participants were excluded if they met any of the following criteria: (1) did not complete three consecutive health examinations between 2006 and 2010; (2) had missing data for any of the CVAI-related variables, including age, BMI, WC, triglyceride (TG), and high-density lipoprotein-cholesterol (HDL-C) in the three examinations; (3) had a history of cancer at baseline in 2006 or developed cancer between 2006 and 2010; (4) had missing data for any of the following covariates: age, sex, reported income, education level, marital status, smoking status, drinking status, physical activity, family history of malignancy, total cholesterol (TC), high-sensitivity C-reactive protein (hs-CRP), and low-density lipoprotein-cholesterol (LDL-C). Finally, a total of 44,213 participants were included in this study (**Figure 1**).

### 2.2 Collection and definitions of covariates

Participants underwent physical examinations conducted by trained medical staff, including measurements of height, weight, and WC. BMI was calculated as weight (kg) divided by height squared (m²). After fasting for at least 8 hours, each participant underwent an ultrasound examination of the liver, gallbladder, and other organs by a trained sonographer. Diagnosis of cirrhosis, fatty liver, gallstones, and gallbladder polyps was made according to pre-established clinical criteria (17, 18).

Blood samples were collected from all participants in the morning after an overnight fast. A total of 5 mL of peripheral venous blood was drawn by trained medical staff and analyzed on a Hitachi 747 automatic analyzer (Hitachi, Tokyo, Japan) to determine serum concentrations of TG, TC, HDL-C, LDL-C, and hs-CRP. Serum TC and TG were measured using enzymatic colorimetric methods, while hs-CRP was measured using a high-sensitivity turbidimetric assay.

Participants completed a standardized questionnaire to collect data on age, gender, reported household income, education level, marital status, smoking status, drinking status, physical activity, and family history of malignancy. Smokers were defined as those who reported smoking at least one cigarette per day, on average, for at least 6 months in the past year. Drinkers were defined as those who reported consuming at least 100 ml of alcohol per day, on average, for at least 6 months in the past year. Physical activity was defined as engaging in at least 30 minutes of exercise per session, at least 3 times per week.

### 2.3 Assessment of Chinese visceral adiposity index

The CVAI calculation formula is (13):


(1) CVAI(man)= −267.93+22.00×LgTG +0.68×age+ 0.03×BMI+4.00×WC−16.32×HDL−C;

(2) CVAI(woman)=−187.32+39.76×LgTG+1.71×age+4.23×BMI+1.12×WC−11.66×HDL−C.

The cumCVAI calculation formula is (19):[(CVAI_2006_+CVAI_2008_)/2×time_1-2_]+ [(CVAI_2008_+CVAI_2010_)/2×time_2-3_], That is the weighted sum of the mean CVAI obtained at each examination, where CVAI_2006_, CVAI_2008_, and CVAI_2010_ represent the CVAI obtained at the examinations in 2006, 2008, and 2010, respectively, and time_1–2_ and time_2–3_ represent the time intervals between consecutive examinations.

### 2.4 Outcome ascertainment

The follow-up period started from the physical examination in 2010 and ended with the occurrence of new-onset GI cancers. For participants who did not develop GI cancers, the follow-up ended on December 31, 2021. For those who died without developing GI cancers, the follow-up ended at the time of death. According to the International Classification of Diseases, Tenth Revision (ICD-10), this study included the following GI cancers: liver cancer (C22), gallbladder or extrahepatic bile duct cancer (C23 and C24), stomach cancer (C16), pancreatic cancer (C25), small bowel cancer (C17), esophageal cancer (C15), and colorectal cancer (C18-C21). Diagnosis was confirmed through the following methods: (1) conducting a physical examination and questionnaire survey for participants every two years, with a cutoff date of December 31, 2021; (2) annually querying relevant medical records from the municipal healthcare system and the Social Security Information System of Kailuan General Hospital; (3) reviewing death certificates from the provincial vital statistics office to obtain additional missing information. These three methods maximize the collection of all cancer cases and minimize omissions. All cancer cases required a definitive pathological diagnosis for re-confirmation. In the absence of a pathological diagnosis, the cases were further evaluated by two or more specialized oncologists. Only when the physicians reached a consensus on the cancer diagnosis would the case be confirmed and included in the cohort.

### 2.5 Statistical analysis

Data analysis was performed using SAS 9.4 statistical software, with a two-sided *P*-value of <0.05 considered statistically significant. Normally distributed continuous data are presented as mean ± standard deviation, and comparisons between groups were conducted using analysis of variance (ANOVA). Skewed continuous data are presented as median and interquartile range, with comparisons between groups performed using the non-parametric Kruskal-Wallis test. Categorical data are expressed as frequencies and percentages, with intergroup comparisons conducted using the chi-square (χ²) test.

The incidence of GI cancers is expressed per 1,000 person-years, and the cumulative incidence of GI cancers was estimated using Kaplan-Meier curves and log-rank tests. A multivariable-adjusted restricted cubic spline (RCS) analysis was conducted to examine the dose-response relationship between CVAI and the risk of GI cancer occurrence. A multivariable Cox proportional hazards regression analysis was conducted to calculate the hazard ratios (HR) and 95% confidence intervals (CI) for the association between cumCVAI quartiles and the incidence of GI cancers. Model 1 represents the univariate analysis; Model 2 adjusts for age and gender based on Model 1; Model 3 further adjusts for TC, hs-CRP, BMI, LDL-C, physical activity, reported income, educational background, marital status, smoking status, drinking status, and family. In multivariate analysis of specific cancer sites, adjustments were made for fatty liver and liver cirrhosis in liver cancer cases, and for gallstones and gallbladder polyps in gallbladder or extrahepatic bile duct cancer cases. Subgroup analyses were conducted stratified by age (<60 years vs. ≥60 years), sex (male vs. female), BMI (<24 kg/m² vs. ≥24 kg/m²), as well as smoking and alcohol consumption status. Finally, several sensitivity analyses were performed to validate the robustness of the study results. We excluded individuals diagnosed with GI cancers within one year of follow-up to eliminate the influence of reverse causation on the findings. Additionally, we excluded participants who were taking statin medications to mitigate the effects of drugs on CVAI levels.

## 3 Results

### 3.1 Baseline characteristics of the participants

This study included a total of 44,213 participants, comprising 34,880 males and 9,333 females, with a mean age of 49.00 ± 11.70 years. Participants were categorized into four groups based on their cumCVAI quartiles. The baseline characteristics of the four groups are presented in **Table 1**, which reveals significant differences in age, sex, TG, TC, HDL-C, LDL-C, WC, BMI, marital status, current drinking status, current smoking status, reported income, educational background (high school graduation or above), sedentary lifestyle, physical exercise, salt intake, non-alcoholic fatty liver disease (NAFLD), liver cirrhosis, and gallstone disease (*P <*0.05). However, no significant differences were observed in the prevalence of gallbladder polyps (*P >*0.05).

**Table 1:** Baseline characteristics of participants by cumCVAI quartiles.

Variables	Q1	Q2	Q3	Q4	*P*-value
N	11053	11053	11054	11053	<0.001
Age (year)	42.15 ± 10.53	47.67 ± 10.37	50.95 ± 10.57	55.24 ± 11.24	<0.001
Male (%)	7590 (68.66)	8933 (80.81)	9173 (82.98)	9184 (83.09)	<0.001
TG (mmol/L)	1.17 ± 0.91	1.60 ± 1.26	1.89 ± 1.48	2.18 ± 1.64	<0.001
TC (mmol/L)	4.74 ± 1.06	4.87 ± 1.21	5.01 ± 1.19	5.10 ± 1.12	<0.001
HDL-C (mmol/L)	1.60 ± 0.38	1.57 ± 0.38	1.53 ± 0.39	1.47 ± 0.37	<0.001
LDL-C (mmol/L)	2.32 ± 0.78	2.38 ± 0.86	2.36 ± 0.92	2.31 ± 0.97	<0.001
Hs-CRP (mg/L)	0.40 (0.16-1.14)	0.60 (0.22-1.60)	0.79 (0.31-2.10)	1.20 (0.50-3.10)	<0.001
WC (cm)	77.79 ± 7.64	84.47 ± 6.71	88.79 ± 7.03	95.43 ± 8.82	<0.001
BMI (kg/m²)	22.27 ± 2.57	24.48 ± 2.62	25.85 ± 2.76	27.84 ± 3.23	<0.001
Marital status (Married,%)	10,387 (93.97)	10,597 (95.87)	10,594 (95.83)	10,532 (95.28)	<0.001
Current drinker (%)	1715 (15.51)	2150 (19.45)	2157 (19.51)	2027 (18.33)	<0.001
Current Smoker (%)	3356 (30.36)	3566 (32.26)	3532 (31.95)	3218 (29.11)	<0.001
Reported income (≥800¥,%)	1413 (12.78)	1301 (11.77)	1394 (12.61)	1561 (14.12)	<0.001
High school graduation or above (%)	2022 (18.29)	2177 (19.69)	1790 (16.19)	1757 (15.89)	<0.001
Sedentary lifestyle (%)					<0.001
<4 h/day	8218 (74.35)	8464 (76.58)	8581 (77.64)	8458 (76.52)	
4–8 h/day	2493 (22.55)	2014 (18.23)	2177 (19.70)	2305 (20.85)	
>8 h/day	342 (3.10)	275 (2.49)	296 (2.66)	290 (2.63)	
Physical exercise (%)					<0.001
Never	1221 (11.05)	1080 (9.77)	960 (8.68)	876 (7.93)	
Occasionally	8730 (78.98)	8674 (78.48)	8368 (75.71)	8021 (72.57)	
Regularly	1102 (9.97)	1299 (11.75)	1726 (15.61)	2156 (19.50)	
Salt intake (%)					<0.001
<6 g/day	1058 (9.57)	997 (9.02)	1025 (9.27)	996 (9.01)	
6–10 g/day	8997 (81.40)	8981 (81.25)	8808 (79.69)	8709 (78.79)	
>10 g/day	998 (9.03)	1075 (9.73)	1221 (11.04)	1348 (12.20)	
NAFLD (%)	1897 (17.16)	2080 (18.82)	2689 (24.31)	2951 (25.67)	<0.001
Liver cirrhosis (%)	9 (0.09)	18 (0.18)	14 (0.13)	17 (0.16)	0.012
Gallstone disease (%)	129 (1.17)	177 (1.60)	182 (1.65)	171 (1.55)	<0.001
Gallbladder polyp (%)	80 (0.73)	68 (0.62)	94 (0.85)	120 (1.09)	0.125

TG, triglyceride; TC, total cholesterol; HDL-C, Highdensity lipoprotein cholesterol; LDL-C, low-density lipoprotein-cholesterol; hs-CRP, high-sensitivity C-reactive protein; WC, waist circumference; BMI, body mass index.

### 3.2 Association between cumulative CVAI and the risk of GI cancers

**Figure 2:**
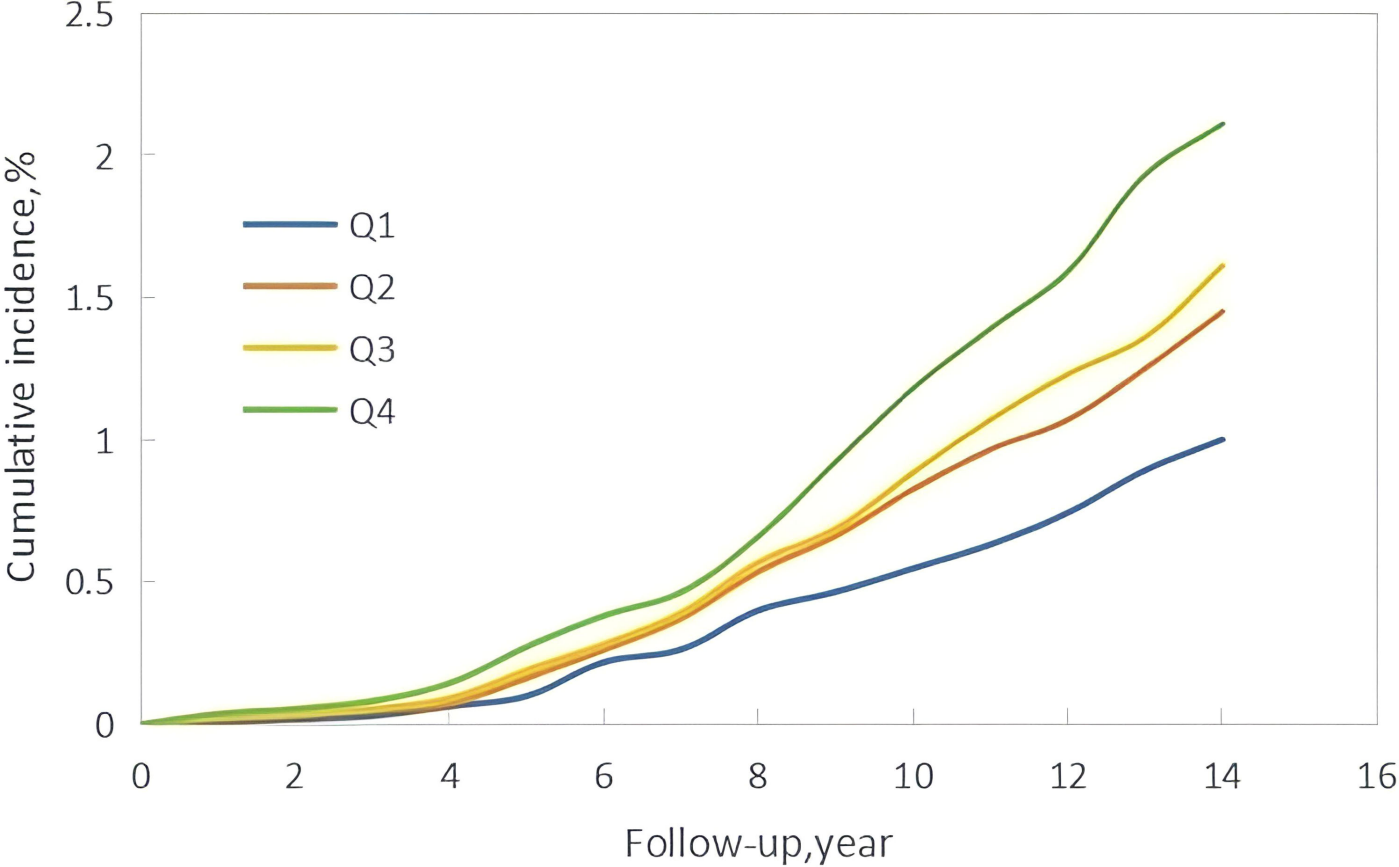
Kaplan–Meier incidence rate of GI cancers by cumCVAI.

**Figure 3:**
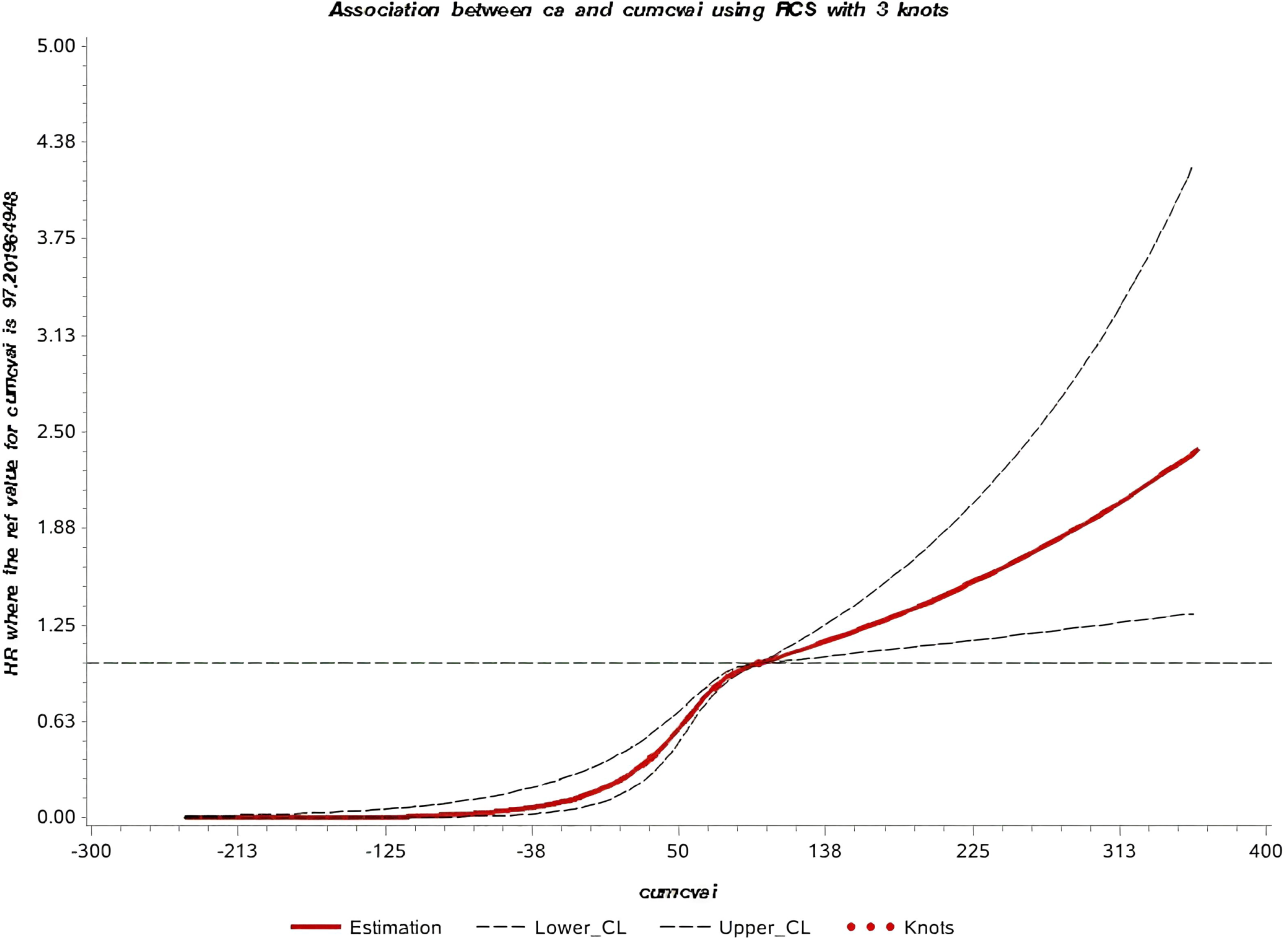
Restricted cubic spline of the association between cumCVAI and the risk of GI cancers.

The median follow-up time in this study was 10.57 ± 1.72 years, during which 760 new cases of GI cancers were identified. These included 74 cases of esophageal cancer, 156 of gastric cancer, 20 of small intestine cancer, 281 of colorectal cancer, 158 of liver cancer, 21 of gallbladder or extrahepatic bile duct cancer, and 50 of pancreatic cancer. Kaplan-Meier curves showed a progressive increase in GI cancers incidence from Q1 to Q4, with statistically significant differences in cumulative incidence across the groups (*P* < 0.001) (**Figure 2**). Additionally, the restricted cubic spline (RCS) curve indicated a nonlinear relationship between cumCVAI and GI cancers events (*P* for overall trend < 0.001; *P* for nonlinear trend < 0.001) (**Figure 3**).

The multivariable analysis of the association between cumCVAI quartiles and the risk of GI cancers, along with incidence rates, is presented in **Table 2**. The incidence rates for GI cancers in the Q1 to Q4 groups were 1.00, 1.45, 1.62, and 2.11 per 1,000 person-years. Compared to the Q1 group, the risk of GI cancers was significantly elevated in the Q2 group (*HR* 1.26; 95% *CI* 1.01, 1.58), Q3 group (*HR* 1.31; 95% *CI* 1.05, 1.64), and Q4 group (*HR* 1.48; 95% *CI* 1.19, 1.85) in Model 3. In the multivariable analysis for specific cancer sites, the risk of cancer was significantly elevated in the gastric cancer Q4 group (*HR* 1.86; 95% *CI* 1.16, 2.99), colorectal cancer Q2 group (*HR* 1.29; 95% *CI* 1.09, 1.66), Q3 group (*HR* 1.34; 95% *CI* 1.02, 1.96), and Q4 group (*HR* 1.44; 95% *CI* 1.07, 2.13), as well as in the liver cancer Q3 group (*HR* 1.49; 95% *CI* 1.01, 2.51) and Q4 group (*HR* 1.61; 95% *CI* 1.05, 2.72). However, no significant associations were found for esophageal cancer, small intestine cancer, gallbladder or extrahepatic bile duct cancer, or pancreatic cancer (**Supplementary Table S1**).

**Table 2:** Association of cumCVAI with GI cancers.

Outcome	Quartile1	Quartile2	Quartile3	Quartile4	P for trend
Case/Total	127/11053	185/11053	199/11054	249/11053	
Incidence rate^a^	1.00	1.51	1.62	2.11	
Model1	1(Reference)	1.52(1.21,1.90)	1.55(1.24,1.94)	2.05(1.65,2.53)	<0.001
Model2	1(Reference)	1.32(1.05,1.66)	1.36(1.09,1.70)	1.61(1.29,2.00)	<0.001
Model3	1(Reference)	1.26(1.01,1.58)	1.31(1.05,1.64)	1.48(1.19,1.85)	<0.001

Model 1: Univariate analysis.

Model 2: Adjusted for age and sex based on model 1.

Model 3: Further adjusted for TC, hs-CRP, BMI, LDL-C, family income, educational background, marital status, smoking status, drinking status, sedentary lifestyle, physical activity, family history of cancer.

Incidence rate^a^: per 1000 person-years.

### 3.3 Association between baseline CVAI and the risk of GI cancers

Based on baseline CVAI quartiles, participants were divided into four groups. The Kaplan-Meier curve indicated that the incidence of GI cancers progressively increased from the Q1 to Q4 groups, with statistically significant differences in cumulative incidence rates between the groups (p < 0.001) (**Supplementary Figure S1**). After adjusting for confounding factors, the risk of GI cancers was significantly higher in the Q2 group (*HR* 1.29; 95% *CI* 1.03, 1.62), Q3 group (*HR* 1.36; 95% *CI* 1.09, 1.70), and Q4 group (*HR* 1.59; 95% *CI* 1.28, 1.97) compared to the Q1 group (**Supplementary Table S2**).

### 3.4 Results of the subgroup and sensitivity analyses

**Figure 4:**
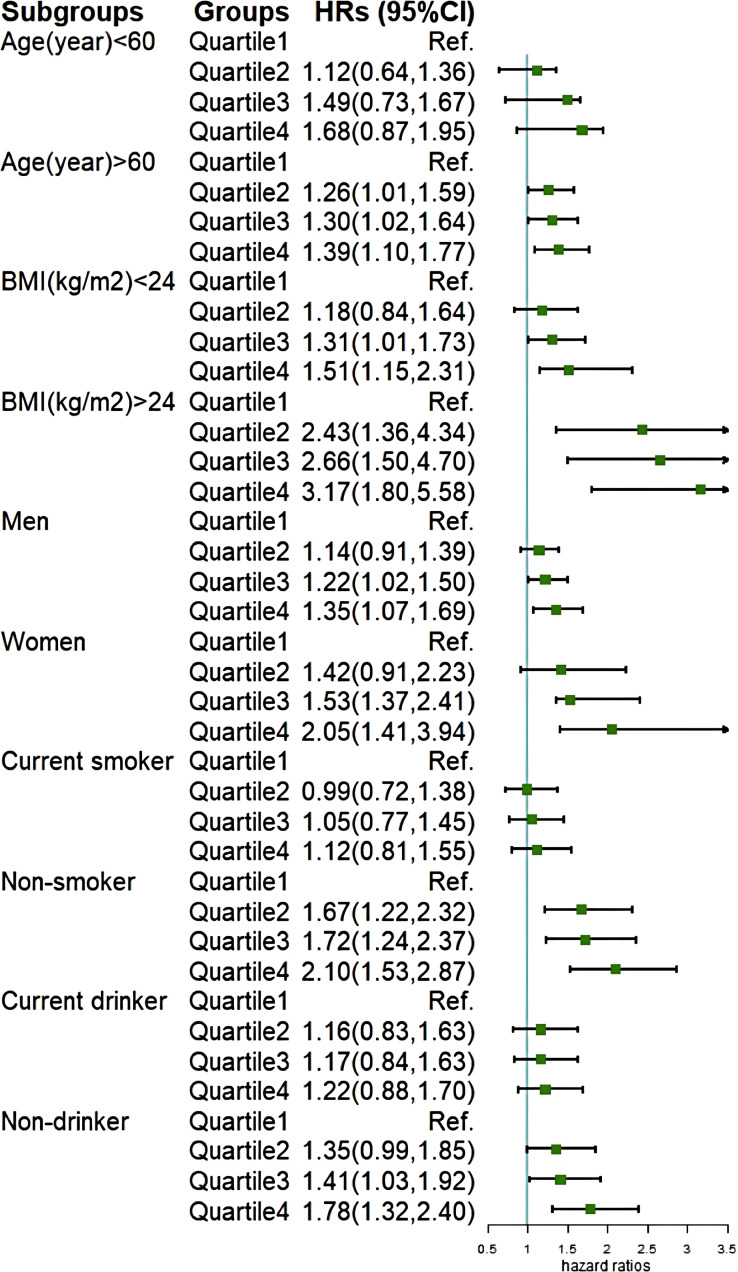
Subgroup analysis of the association of cumCVAI with the risk of GI cancers.

We conducted stratification based on participants’ age, gender, BMI, smoking status, and drinking status (**Figure 4**), and found no significant interactions with cumCVAI (p > 0.05). Except for younger individuals, current smokers, and current drinkers, all other subgroups demonstrated a significant increase in the risk of GI cancers with higher quartiles of cumCVAI. In sensitivity analyses, we excluded 117 participants who developed GI cancers within one year of follow-up (**Supplementary Table S3**) and 372 participants taking lipid-lowering medications (**Supplementary Table S4**). The results showed no significant changes, and the association between cumCVAI and the risk of GI cancers remained significant.

## 4 Discussion

In this prospective cohort study based on the Kailuan cohort, we found that cumCVAI is a risk factor for new-onset GI cancers, with the risk of GI cancers gradually increasing as cumCVAI quartiles rise. Furthermore, the RCS curve indicates a clear dose-response relationship between cumCVAI and the incidence of GI cancers. In the multivariable analysis of specific cancer sites, high cumCVAI was associated with an increased risk of gastric cancer, colorectal cancer, and liver cancer. Subgroup analyses and sensitivity analyses did not alter the original conclusions, further validating the robustness of the findings in this study.

Globally, the number of obese individuals exceeds 2 billion, with a significant increase in obesity rates across Asia. As early as 2014 (20), China became the country with the highest number of obese individuals in the world, accounting for 14% of the global obese population (21). BMI is the most commonly used surrogate measure for obesity; however, it is more applicable to overall obesity and does not reflect the relationship between fat and lean body mass, nor does it provide insights into fat distribution. Compared to fat in other areas, VAT poses greater harm to the human body (22). Excessive VAT can lead to metabolic disorders and an inflammatory state, promoting the onset and progression of cancer (23). Increasing evidence suggests that VAT is a risk factor for various types of cancer (24).

Due to its ease of acquisition, CVAI serves as a good surrogate marker for VAT in the Chinese population. Previous studies have not reached a definitive conclusion regarding the relationship between CVAI and overall gastrointestinal cancers. Our research indicates that high cumCVAI significantly increases the incidence of gastrointestinal cancers, with VAT being a critical factor in this association. A cohort study from the United States demonstrated that, after adjusting for confounding factors, VAT is a risk factor for cancer, a finding consistent with our results, particularly in the male subgroup. However, no association was observed in the female population (25). A similar study from the United States, which included both White and Black participants, found a positive association between VAT and cancer incidence, with conclusions independent of racial differences (26). The relationship between CVAI and GI cancers can be attributed to several factors. Firstly, adipose tissue releases inflammatory factors, leading to a chronic inflammatory state in the body (27). There is evidence suggesting that VAT exerts a stronger pro-inflammatory effect than subcutaneous adipose tissue (SAT), thereby leading to enhanced metabolic activity in the body (28), the release of a greater number of cytokines leads to DNA damage (29), impacting DNA synthesis and repair (30), which increases the mutation rate and facilitates the transformation of normal cells into cancerous cells. What’s more, obesity is directly associated with insulin resistance, which stimulates compensatory insulin secretion, leading to hyperinsulinemia (31). Insulin activates intracellular signaling pathways, thereby promoting accelerated growth and increased invasiveness of tumor cells (32). A prospective cohort study conducted in China similarly indicated a significant positive correlation between elevated CVAI and the risk of developing diabetes (33), Diabetes is a known risk factor for GI cancers (34, 35), further corroborating the findings of our study.

We found that high cumCVAI is associated with an increased risk of gastric cancer, colorectal cancer, and liver cancer. Previous studies on the impact of visceral fat on colorectal cancer have yielded inconsistent conclusions. A cross-sectional study conducted among East Asian populations indicated that participants with a visceral fat area exceeding 136.6 cm² had a significantly elevated risk of colorectal cancer (*OR* = 4.07, 95% *CI* 1.01–16.43, *P* = 0.03) (36). Similarly, a dose-response meta-analysis indicated that for every 25 cm² increase in VAT, the risk of developing colorectal adenomas increased by 13% (37), Colorectal adenomas are precursors to colorectal cancer (38). However, a case-control study by Elife Erarslan et al. found that visceral fat accumulation does not increase the risk of colorectal cancer (39). Previous studies on the impact of visceral fat on gastric and liver cancers have yielded results that are generally consistent with ours. A case-control study from Japan indicated a positive correlation between visceral fat area and the incidence of gastric cancer, while BMI showed a negative correlation with gastric cancer incidence. Furthermore, when the visceral fat area was ≥100 cm², the risk of developing gastric cancer significantly increased, with an *OR* (95% *CI*) of 1.96 (1.02-3.76) (40). A Mendelian randomization study based on the UK Biobank and the Adult Health and Aging Genetic Epidemiology Study cohort found that liver fat and VAT are associated with an increased risk of primary liver cancer, with VAT showing a higher predictive value than traditional obesity measures such as BMI and WC (41). However, we did not find that high cumCVAI increases the risk of esophageal cancer, small intestine cancer, gallbladder and extrahepatic bile duct cancer, or pancreatic cancer, indicating the need for further research to explore this issue.

Subgroup analysis further validated our findings. In the gender-specific analysis, we found that high cumCVAI was more strongly associated with gastrointestinal cancers in females compared to males. Research has indicated that women are more prone to central obesity, and there are differences in visceral fat distribution between genders (42). At the same time, G. Boden et al. found that elevated plasma free fatty acid (FFA) lead to insulin resistance (43), and the clearance rate of FFA in females is 64% higher than that in males (44). Therefore, an increase in CVAI among women can be regarded as a warning sign, prompting timely intervention measures. We also observed that the predictive capability of CVAI is stronger in non-smoking and non-drinking populations. This may be attributed to the fact that both smoking and drinking are recognized carcinogenic factors (45–47), which could potentially obscure the impact of CVAI on gastrointestinal malignancies.

Our study has several strengths: (1) it offers a unique perspective on the risk factors for GI cancers; (2) it is based on the Kailuan cohort, which has a large sample size, relatively long follow-up duration, a wide age range among participants, and an almost zero loss to follow-up rate; (3) it extensively evaluated potential confounding factors, including lifestyle habits and family history of cancer. However, certain limitations should also be noted: (1) information on smoking, drinking, physical activity, and sedentary habits was self-reported by participants, which may introduce recall bias; (2) while endoscopy is the gold standard for diagnosing gastric and colorectal cancers, participants were not subjected to this procedure due to budget constraints within the Kailuan cohort, potentially leading to missed asymptomatic cancer cases; (3) the cohort exhibits an uneven gender distribution, with a higher proportion of males, given the industrial nature of the Kailuan Group; (4) the Kailuan cohort is primarily based on an urban population in northern China, which may not be representative of the entire Chinese population.

## 5 Conclusions

This study demonstrates a positive correlation between high cumCVAI and the incidence of GI cancers, particularly among participants with older adults, women, BMI ≥ 24, non-smoker and non-drinker. Therefore, monitoring dynamic changes in CVAI may provide a theoretical basis for the prevention of GI cancers.

## Data Availability

The raw data supporting the conclusions of this article will be made available by the authors, without undue reservation.
