# Supplementary material for: Association of cumulative exposure to Chinese visceral adiposity index and gastrointestinal cancer: a prospective cohort study
Source: Front Oncol. 2025 Jul 3;15:1534682. doi: 10.3389/fonc.2025.1534682 (PMC12267042; doi:10.3389/fonc.2025.1534682)
Supplement: Supplementary file 1 [file DataSheet1.docx]

**Additional file 1**

**Additional tables**

**TABLE S1** Association of cumCVAI with the risk of specific site of GI cancers.

**Fig S1** Kaplan–Meier incidence rate of GI cancers by baseline CVAI.

**TABLE S2** Association of baseline CVAI with GI cancers.

**TABLE S3** Association of cumCVAI with GI cancers exclude participants who occurred GI cancers within the 1st year.

**TABLE S4** Association of cumCVAI with GI cancers exclude participants who took statins.

**TABLE S1.** Association of cumCVAI with the risk of specific site of GI cancers.

| **Cancer type** | **Cases** | **Quartile 1** | **Quartile 2** | **Quartile 3** | **Quartile 4** |
| --- | --- | --- | --- | --- | --- |
| **Esophageal cancer** | 74 | 1(Reference) | 1.03(0.49,2.16) | 1.66(0.86,3.21) | 1.44(0.70,2.95) |
| **Stomach cancer** | 156 | 1(Reference) | 1.08(0.65,1.80) | 1.21(0.74,1.89) | **1.86(1.16,2.99)** |
| **Small intestine cancer** | 20 | 1(Reference) | 1.20(0.20,7.21) | 1.74(0.33,9.11) | 2.88(0.60,13.89) |
| **Colorectal cancer** | 281 | 1(Reference) | **1.29(1.09,1.66)** | **1.34(1.02,1.96)** | **1.44(1.07,2.13)** |
| **Liver cancer^a^** | 158 | 1(Reference) | 1.39(0.84,2.30) | **1.49(1.01,2.51)** | **1.61(1.05,2.72)** |
| **Gallbladder or extrahepatic bile duct cancer^b^** | 21 | 1(Reference) | 1.61(0.29,8.81) | 2.54(0.52,12.45) | 2.69(0.55,13.33) |
| **Pancreatic cancer** | 50 | 1(Reference) | 0.75(0.29,1.90) | 1.03(0.42,2.49) | 1.42(0.62,3.28) |

All models were adjusted for age, sex, TC，hs-CRP，BMI，LDL-C，family income，educational background，marital status，smoking status，drinking status，sedentary lifestyle，physical activity，family history of cancer.

^a^Liver cancer: Further adjusted for liver cirrhosis and fatty liver disease.

^b^Gallbladder or extrahepatic bile duct cancer :Further adjusted for gallstone disease.

**Fig S1.** Kaplan–Meier incidence rate of GI cancers by baseline CVAI.


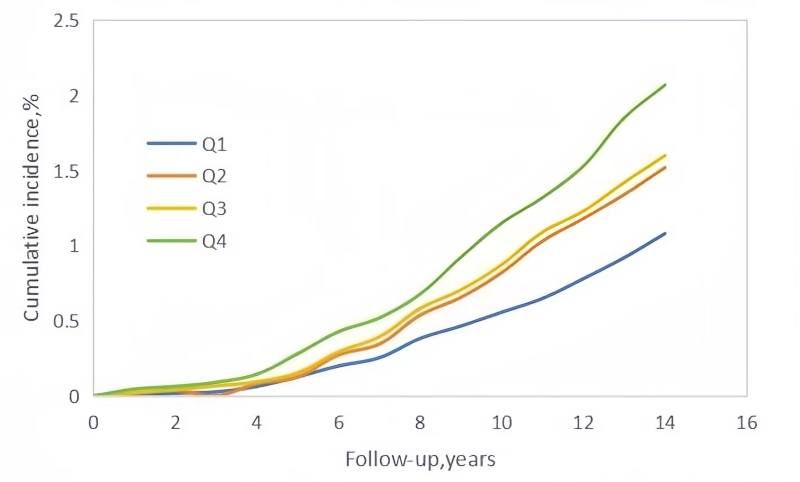


**TABLE S2.** Association of baseline CVAI with GI cancers.

|  | Quartile1 | Quartile2 | Quartile3 | Quartile4 | P for trend |
| --- | --- | --- | --- | --- | --- |
| Case/Total | 131/11053 | 184/11053 | 200/11054 | 245/11053 |  |
| Incidence rate^a^ | 1.08 | 1.52 | 1.60 | 2.07 |  |
| Model1 | 1(Reference) | 1.41(1.13,1.77) | 1.56(1.25,1.94) | 1.96(1.58,2.42) | ＜0.001 |
| Model2 | 1(Reference) | 1.35(1.08,1.69) | 1.47(1.18,1.83) | 1.79(1.45,2.22) | ＜0.001 |
| Model3 | 1(Reference) | 1.29(1.03,1.62) | 1.36(1.09,1.70) | 1.59(1.28,1.97) | ＜0.001 |

Model 1：Univariate analysis.

Model 2：Adjusted for age and sex based on model 1.

Model 3：Further adjusted for TC、hs-CRP、BMI、LDL-C、family income，educational background，marital status，smoking status，drinking status，sedentary lifestyle，physical activity，family history of cancer.

Incidence ratea^a^: per 1000 person-years.

**TABLE S3.** Association of cumCVAI with GI cancers exclude participants who occurred GI cancers within the 1st year.

|  | Quartile1 | Quartile2 | Quartile3 | Quartile4 | P for trend |
| --- | --- | --- | --- | --- | --- |
| Case/Total | 110/11036 | 161/11022 | 166/11028 | 206/11010 |  |
| Incidence rate^a^ | 0.85 | 1.26 | 1.38 | 1.72 |  |
| Model1 | 1(Reference) | 1.50(1.18,1.87) | 1.53(1.21,1.90) | 2.00(1.63,2.49) | ＜0.001 |
| Model2 | 1(Reference) | 1.31(1.04,1.61) | 1.35(1.09,1.72) | 1.58(1.21,1.93) | ＜0.001 |
| Model3 | 1(Reference) | 1.22(1.01,1.56) | 1.32(1.03,1.67) | 1.46(1.15,1.86) | ＜0.001 |

Model 1：Univariate analysis.

Model 2：Adjusted for age and sex based on model 1.

Model 3：Further adjusted for TC、hs-CRP、BMI、LDL-C、family income，educational background，marital status，smoking status，drinking status，sedentary lifestyle，physical activity，family history of cancer.

Incidence ratea^a^: per 1000 person-years.

**TABLE S4.** Association of cumCVAI with GI cancers exclude participants who took statins.

|  | Quartile1 | Quartile2 | Quartile3 | Quartile4 | P for trend |
| --- | --- | --- | --- | --- | --- |
| Case/Total | 127/11030 | 189/11004 | 190/10956 | 244/10851 |  |
| Incidence rate^a^ | 1.01 | 1.53 | 1.60 | 2.12 |  |
| Model1 | 1(Reference) | 1.55(1.23,1.94) | 1.61(1.24,1.96) | 2.09(1.68,2.73) | ＜0.001 |
| Model2 | 1(Reference) | 1.37(1.05,1.78) | 1.39(1.08,1.81) | 1.64(1.31,2.07) | ＜0.001 |
| Model3 | 1(Reference) | 1.24(1.01,1.58) | 1.30(1.03,1.63) | 1.47(1.18,1.89) | ＜0.001 |

Model 1：Univariate analysis.

Model 2：Adjusted for age and sex based on model 1.

Model 3：Further adjusted for TC、hs-CRP、BMI、LDL-C、family income，educational background，marital status，smoking status，drinking status，sedentary lifestyle，physical activity，family history of cancer.

Incidence ratea^a^: per 1000 person-years.
